# Assessing success—a commentary on the necessity of outcomes measures

**DOI:** 10.1186/s13011-015-0017-2

**Published:** 2015-05-14

**Authors:** Ruchi M. Sanghani, Alexandra L. Carlin, Alexander K. Moler

**Affiliations:** The Coalition Against Drug Abuse, 900 Broadway #704, New York, NY 10003 USA; Department of Psychology, Fordham University, 441 E. Fordham Road, Dealy Hall 226, Bronx, NY 10458 USA

**Keywords:** Outcomes measurements, Quality assessment, Standardization, Oversight, Success metrics

## Abstract

Measurements for outcomes reporting are not fully formed and utilized in the American addiction industry, though formulated and adopted elsewhere in the world. While studies have established demographic information about those needing and receiving treatment as well as the facilities that offer such treatment, short- and long-term outcomes are scantily reported. This commentary serves as a call to action to developing such metrics in the US by illustrating the benefits to treatment providers and clients of creating outcomes standards, and the subsequent improvements in quality of care needed to reach those standards. Benefits of developing these metrics beyond improved quality of care may also include a more efficient allocation of resources, such as time and money. Additionally, the delivery of more effective, personalized, and outcomes-driven addiction treatment may increase client buy-in and foster a more open communication channel between clients and providers during and after treatment.

## Background

Recent data from the Substance Abuse and Mental Health Services Administration’s (SAMHSA’s) National Survey on Drug Use and Health [1] (NSDUH) indicates that 23.5 million Americans aged 12 or older (approximately 9.4 % of the American population over age 12) are current illicit drug users. Of these illicit drug users, 11.2 % receive treatment for substance use disorders and addiction [1]. According to SAMHSA’s National Survey of Substance Abuse Treatment Services [2] (N-SSATS), 14,311 addiction treatment facilities or centers in the US provided care for 1.25 million clients in 2012. 54.9 % of admitted cases, in one national survey, have had at least one prior treatment episode [3]. While demographic data about those struggling with addiction are readily reported [1, 3, 4], less information is available on those who have completed or exited a treatment facility. SAMHSA’s Treatment Episodes Data Set [3] (TEDS) reports on number of prior treatment episodes upon entry into a treatment center; however indicators of treatment effectiveness and follow-up data are not explicitly and regularly tracked on a national level. Furthermore, TEDS (and other) surveys do not make unique cases publicly available further highlighting this lack of information, as well as exposing a missed opportunity to qualitatively evaluate individualized treatment plans.

## Main text

Standardized tools for addiction professionals to measure short- and long-term outcomes and success, across multiple metrics, are not readily available in the US. In fact, the definition of “success” as it pertains to recovery is, at best, vaguely defined. For some, including 12-Step adherents, “success” is complete abstinence from any substance, whereas others may consider “success” to include the use of pharmacotherapies to manage symptoms and cravings, or employing techniques to use a particular substance responsibly [5]. Further still, the concept of “recovery” is ill-defined, and is generally poorly understood [6]. Peer reviewed studies on “success” and outcomes lack universal consensus for the American addiction treatment system [7], and are limited in their practical application. For example, the prominent TEDS-D survey defines “success” solely as completing a treatment program. While more comprehensive outcomes measures have been investigated on facility [8] and state [9, 10] levels, long-term, multi-metric measures on a national scale have seldom been investigated, particularly with support, financial or otherwise, from governing bodies. Analogous metrics have, however, been created for addiction treatment systems elsewhere. The European Union, with support from the United Nations, has published extensive survey tools and guidelines for addiction treatment providers to measure and assess outcomes [11–13]. Similarly, the Australian government provides metrics and compulsory standards, as well as toolkits containing guidelines, resources, and educational materials [14].

Relapse rate has long been the standard indicator by which recovery success is measured within the industry. Historically though, documentation of this metric has been inconsistent, and has relied heavily on estimated figures. Estimates suggest that as many as 80 % of those who seek and complete addiction treatment will relapse after treatment termination [15]. These grim statistics suggest, perhaps, that relapse rate cannot serve as the sole indicator of recovery success, lest the industry as a whole be interpreted as widely ineffectual. Addiction treatment and recovery are multifaceted processes, and treatment success cannot be determined by a single metric. While relapse rates may indicate some facet of success, other metrics, such as comorbidities; personal, legal, and emotional state; and external conditions, may prove significant to long-term recovery success and should be considered as potential metrics to determining a composite success variable [16].

The currently available data on post-rehabilitation outcomes is inconsistent, and demonstrates a lack of reporting in the addiction space, which can contribute to two major consequences. First, without accurate outcomes evaluation, facilities offering poor quality of care are able to continue offering such treatment without regular evaluation. This absence of measurements and tracking also results in a poor allocation of resources: facilities offering poor treatment quality continue to consume resources that would otherwise be better spent by facilities engaging in ethical, high quality treatment. Secondly, without outcomes measurements, there are fewer options for conducting quality improvement initiatives, both on the facility level and on a broad level throughout the space. This stagnates the industry, as there are no quantifiable standards to achieve and surpass; rather, progress becomes more ideological and thus, harder to define. To motivate constant quality improvement, measurable outcomes must be assessed and publicly reported.

Outcomes are directly dependent on the level of care received throughout treatment. By setting outcomes standards, the quality of treatment may elevate, and the form of treatment delivery may change to meet these standards. Dialogue on treatment methodologies is widespread in the addiction space, often focused on which particular treatment method is the most effective. Following the evidence borne from current trends in the broader American healthcare system, this dialogue should include personalized treatment plans [17, 18]. Designing and implementing outcomes reporting, based on leading treatment methodologies, would, in effect, install those methodologies into facilities’ practices.

An outcomes reporting system that incorporates leading treatment methodologies could have profound effects on the treatment space. Through delivering more effective outcomes-driven treatment, with standards that allow for individualization, clients may engage in building a treatment plan that is best suited to their needs, preferences, and belief system [17]. This possibility may increase buy-in from clients, who would otherwise be resistant to receiving treatment without a more trust-founded and personal connection to the treatment plan and provider – ultimately culminating in increased treatment adherence, setting the client up for long-term success [17–20]. By involving clients in the development of their own treatment plan, clients may feel more empowered and profoundly engaged with the recovery process. The clients become active participants, rather than resigned recipients of a rote prescription for an immutable treatment process.

The ways in which outcomes are measured may also benefit post-treatment client buy-in. Previous clients, who struggle or relapse after receiving treatment, may be difficult to reach for follow-up. These past clients may feel guilt, shame, or defeat, thus prompting them to distance themselves from their previous treatment facility [21]. A personal treatment plan may make the client feel more connected to the treatment provider; therefore, opening the communication channel between alumni and treatment centers to follow-up or receive additional help.

Increasing treatment effectiveness also requires assessing aspects of treatment beyond what is considered clinical. Administrative assessments must be conducted to determine which services a treatment provider is equipped to provide. Attention must be drawn to facilities’ physical accommodations and the abilities of the staff, including credentials, legal limitations, and staff-to-client ratios. Accurate and truthful assessments of these capabilities will allow for facilities to provide only the treatment plans they can manage, rather than overextending themselves and causing unnecessary expenditures. Treatment providers must also consider who is an ideal client for their center – one whose needs align best with the providers’ missions, skills, and services. Ideal matches allow for the right treatment to be delivered at the right time with the right resources to the right people. Doing so allows for a much more efficient allocation of resources, which saves clients and treatment providers time, money, and personnel.

## Conclusion

Ultimately, more effective treatment driven by smarter decision-making and outcomes standards has the capability to benefit clients and their loved ones, as well as facilities themselves. In addition to the time, money, and other resources saved for both clients and facilities, facilities have the added benefit of using outcomes measures for accreditation, marketing materials, and their reputation in the space. A multimetric system for evaluating outcomes must be developed to motivate enhanced treatment effectiveness, cyclically benefiting individuals and facilities in the long-term. Future projects should identify these metrics using those established in the European Union and Australia as guides, and develop a standardized process by which outcomes can be measured and reported.

## Abbreviations

SAMHSA: Substance abuse and mental health services administration
NSDUH: National survey on drug use and health
N-SSATS: National survey of substance abuse treatment services
TEDS: Treatment episodes data set
